# Dermatomyosite du sujet âgé: étude de 4 observations dans le sud tunisien

**Published:** 2012-10-06

**Authors:** Faten Frikha, Mouna Snoussi, Raida Ben Salah, Noura Saidi, Neila Kaddour, Zouhir Bahloul

**Affiliations:** 1 Service de Médecine interne CHU Hédi Chaker 3029 Sfax, Tunisia

**Keywords:** Myopathie inflammatoire, dermatomyosite, personnes âgées, inflammatory myopathy, dermatomyositis, elderly

## Abstract

La dermatomyosite (DM) touche essentiellement l’adolescent et l’adulte jeune, elle est très rare chez le sujet âgé, le plus souvent associée à des complications iatrogènes et à une pathologie cancéreuse. Nous avons étudié les caractéristiques de la DM du sujet âgé à travers une étude rétrospective dans laquelle nous avons comparé 4 patients âgés de plus de 65 ans au début de la myosite avec 40 sujets jeunes.

## Introduction

Les myopathies inflammatoires (MI) sont des connectivites rares caractérisées par l’association d’un déficit musculaire et la présence d’un infiltrat inflammatoire au sein du muscle squelettique. Les MI regroupent trois entités principales: la polymyosite (PM), la dermatomyosite (DM) et la myosite à inclusions [1, 2]. Elles sont dotées d’un grand polymorphisme clinique et évolutif. Des désordres immunitaires cellulaires et humoraux sont probablement à l’origine de ces connectivites. Des progrès considérables ont été réalisés ces dernières années dans la compréhension de leur pathogénie. La DM se distingue par une atteinte cutanée caractéristique. Elle est caractérisée par une atteinte primitive péri vasculaire de mécanisme humoral [2, 3]. Le diagnostic de cette affection repose sur un faisceau d’arguments cliniques, enzymologiques, électromyographiques et histologiques. La DM touche essentiellement l’adolescent et l’adulte jeune en Tunisie [4], elle est très rare chez le sujet âgé, le plus souvent associée à une pathologie cancéreuse [5]. Le traitement et le pronostic restent comparables. Le but de travail est de déterminer, à travers une étude rétrospective, les caractéristiques épidémiologiques, cliniques, para cliniques, évolutives et les modalités thérapeutiques de quatre patients dont l’âge était supérieur ou égal à 65 ans au moment du diagnostic de la myosite (dermatomyosite) parmi une cohorte de 78 patients atteints de MI (44 cas atteints de DM), suivis au service de médecine interne du CHU Hèdi Chaker de Sfax entre 1979 et 2010 et de les comparer à une population témoin plus jeune.

## Méthodes

Nous rapportons une étude rétrospective incluant 4 patients dont l’âge était supérieur à 65 ans au moment du diagnostic de la DM parmi une cohorte de 44 patients atteints de DM, suivis au service de médecine interne du CHU Hèdi Chaker de Sfax durant une période de 31 ans s’étalant de Janvier 1979 à décembre 2010. Le diagnostic de DM a été retenu en se basant sur les critères de Bohan et Peter [1].

Nous avons étudié les données épidémiologiques (âge, sexe), le mode de début (aigu, subaigu, chronique), le délai du diagnostic ( durée qui sépare la date d’apparition du premier signe de la maladie de la date du diagnostic définitif), les signes cliniques en rapport avec la maladie (signes généraux, cutanés, musculaires, digestifs, pulmonaires, cardiaques, neurologiques et rénaux), les données biologiques ( recherche d’un syndrome inflammatoire, dosage des enzymes musculaires, étude immunologique), l’étude des données de l’électromyogramme (EMG) à la recherche d’un syndrome myogène et l’étude anatomopathologique (biopsie musculaire et cutanée). Nous avons précisé les modalités thérapeutiques (en précisant les molécules utilisées, leur posologie, la durée du traitement); ainsi que les données évolutives (en se basant sur les éléments cliniques et biologiques).

## Résultats

### Observation 1

Mr A.A. âgé de 84 ans, aux antécédents d’insuffisance cardiaque, était hospitalisé en novembre 2001 pour arthralgies de type inflammatoire touchant les grosses articulations (hanches, genoux et épaules) associées à des troubles de la déglutition avec une dysphagie, des fausses routes itératives évoluant depuis deux mois motivant son hospitalisation. L’examen clinique objectivait un érythème sans œdème siégeant au niveau du visage en périorbitaire et au niveau du décolleté, un déficit musculaire des ceintures scapulaires et pelviennes au testing musculaire. L’examen neurologique révélait des réflexes ostéo-tendineux faibles. Le reste de l’examen clinique était sans particularité. Le bilan biologique montrait un syndrome inflammatoire biologique avec une vitesse de sédimentation (VS) accélérée à 80 mm la première heure, un taux de CRP élevé à 92 mg/l, une hypo albuminémie à 30g/l, une élévation des alpha 1 globulines (3g/l), alpha 2 globulines (10,2g/l) et gamma globulines (15g/l). On notait également une insuffisance rénale avec une urée à 23 mmol/l et une créatinine à 212 umol/l. L’hémogramme retrouvait un taux de leucocytes à 12000 éléments/mm3 avec une hémoglobine à 12,9 g/dl et des plaquettes normales. On notait également une élévation modérée des transaminases avec un taux des ASAT et des ALAT à deux fois la normale, des CPK à 1380 UI/l et des LDH à 1110 UI/l. La recherche d’anticorps anti-nucléaires était positive à 1/80 de type hépatites B et C était négatives. La biopsie musculaire du deltoïde confirmait l’existence d’une dematopolymyosite (DPM) aiguë en montrant des signes histologiques de myopathie inflammatoire avec un infiltrat inflammatoire lympho-histiocytaire dans les régions septales, une inégalité de la taille des fibres des fibres atrophiques arrondies, des fibres en voie de nécrose, absence d’atrophie péri fasciculaire. L’électromyogramme (EMG) montrait des signes d’atteinte neurogène périphérique démyélinisante proximale et distale sensitivomotrice pouvant être en rapport avec une forme sévère de sa DM. Devant ces données, le diagnostic de Dermatomyosite (DM) était retenu selon les critères de Bohan et Peter [1]. L’enquête à la recherche d’une néoplasie profonde associée était négative avec un examen ORL normal, des marqueurs tumoraux (AFP, ACE, CA 19-9) normaux, une radiographie thoracique et une échographie abdominopelvienne normales. Le patient était traité par trois bolus de méthylprednisolone (solumédrol^®^) à la dose de 1 g/jour relayés par une corticothérapie à forte dose (prednisolone 1mg/kg/j soit Solupred^®^ 3 comprimés par jour) avec une amélioration partielle des forces musculaires et des fausses routes et du taux des enzymes musculaires (CPK à 144 UI/l et LDH à 707 UI/l).

### Observation 2

**Figure 1:**
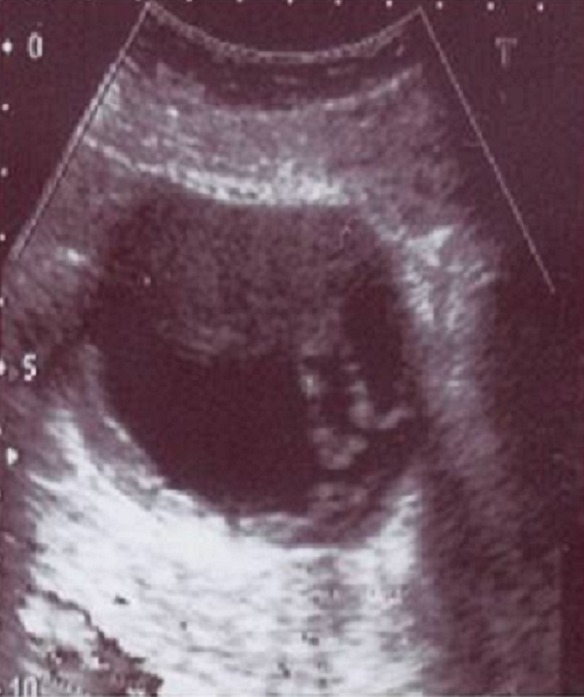
Echographie pelvienne. Masse kystique ovarienne à paroi épaissie renfermant des bourgeonnements tissulaires très suspects de malignité chez une patiente de 86 ans prise en charge pour determatomyosite au service de médecine interne du CHU Hèdi Chaker, Tunisie

Madame K.H. âgée de 86 ans, aux antécédents d’arythmie complète par fibrillation auriculaire depuis 9 ans sous Cordarone et Sintrom, hypothyroïdie sous L-thyroxine, était hospitalisée en juin 2008 pour des myalgies diffuses avec déficit musculaire des deux ceintures, œdème et érythème du visage évoluant depuis trois mois. A l’admission, la patiente était apyrétique, pesait 64 Kg et sa Pression artérielle mesurée à 120/70 mmHg. L’examen clinique trouvait un érythème du visage et en périorbitaire en lunette, des lésions érythémato-squameuses au niveau du décolleté. On objectivait un déficit musculaire des deux ceintures scapulaires et pelviennes avec un score à 63/75 sur l’échelle fonctionnelle des myosites. La biologie montrait une anémie à 9 g/dl hypochrome microcytaire, des leucocytes à 20000 éléments/mm 3, des plaquettes normales, une vitesse de sédimentation à 38 mm la première heure, un taux de CRP à 29 mg/l. on notait également une insuffisance rénale avec un taux d’urée à 13,3 mmol/l et une créatinine à 178 umol/l. Les CPK étaient franchement élevées à 7504 UI/l/. Les transaminases étaient élevées avec un taux d’ASAT et d’ALAT à 387 et 292 UI/l respectivement. La recherche d’anticorps anti-nucléaires était positive à 1/1280 de type homogène. Le bilan thyroïdien était normal. L’électromyogramme (EMG) montrait des signes d’atteinte myogène diffuse avec des potentiels de fibrillation caractéristique d’une polymyosite en phase active. Le diagnostic d’une dermatomyosite DM était alors retenu. Devant l’âge avancé, l’origine secondaire de sa maladie était évoquée. Un bilan à la recherche d’une néoplasie profonde trouvait un taux de CA 125 élevé à 95,1 UI/ml (la valeur normale étant inférieure à 35UI/ml). L’échographie abdominopelvienne montrait une deux masses kystiques sus et latéro-utérine à paroi épaissie renfermant des bourgeonnements tissulaires (Figure 1). Ces masses étaient d’origine vraisemblablement ovarienne mesurant respectivement 5X8X10 cm et 4,8X6,6 cm. Le diagnostic de DM secondaire à une néoplasie ovarienne était alors retenu.

La radiographie thoracique objectivait une cardiomégalie avec une image calcifiée du champ pulmonaire gauche séquellaire sans signe de malignité. Devant le déficit musculaire important et la myolyse sévère avec insuffisance rénale, la patiente était mise sous prednisone à forte dose (1mg/kg/j). L’évolution était marquée par l’amélioration partielle de la myolyse avec un taux de CPK à 3280 UI/l, l’absence d’amélioration du déficit musculaire, l’aggravation de son état respiratoire avec l’installation d’une insuffisance respiratoire aiguë nécessitant son transfert en réanimation, décision que les parents avaient refusé, la patiente était alors mise sortante contre avis médical.

### Observation 3

Madame B.M. âgée de 71 ans, aux antécédents de fracture de la hanche gauche avec mise en place d’une prothèse, était hospitalisée dans le service en Août 2003 pour oedème du visage, des membres supérieures et un érythème orbitaire, le tout évoluant de puis quinze jours. L’examen clinique objectivait des lésions érythémato-maculeuses mal limitées siégeant au niveau des pommettes et des paupières avec œdème à ce niveau, de la face antérieure des deux genoux et jambes. Le reste de l’examen clinique était sans particularité notamment l’absence de déficit musculaire des ceintures avec un signe du tabouret négatif. La patiente signalait la notion d’un acrosyndrome des mais avec à la capillaroscopie un aspect de microangiopathie organique et présence de mégacapillaires. A la biologie, on trouvait une hémoglobine à 11,6 g/dl, des leucocytes et des plaquettes normales. La vitesse de sédimentation était accélérée à 67 mm la première heure, le dosage de la CRP était négatif, les transaminases étaient également normales, les anticorps anti-nucléaires et les anticorps anti-phospholipides étaient négatifs. Le taux des CPK était élevé à 665 UI/l, les LDH étaient à 764 UI/l et les aldolases à 8,22 UI/l. Le diagnostic de DPM était alors évoqué. Un EMG pratiqué concluait à un tracé myogène riche et peu ample au niveau des deltoïdes compatibles avec une DPM peu sévère. La biopsie musculaire montrait la présence de quelques foyers inflammatoires au niveau des septa inter fasciculaire et dans l’endomysium évoquant une myopathie inflammatoire. Un bilan à la recherche d’une néoplasie profonde pratiqué concluait à un dosage normal des marqueurs tumoraux, un examen ORL sans anomalies, une radiographie thoracique montrant un syndrome interstitiel pouvant évoquer une atteinte pulmonaire dans le cadre de sa maladie, une échographie abdominopelvienne, échographie mammaire et fibroscopie oeso-gastro-duodénale étaient normales. L’électrophorèse des protéines sériques montrait la présence d’une fraction anormale migrant en zone gamma confirmée par l’immunoélectrophorèse qui objectivait la présence de chaînes légères kappa et lambda dans le sang et dans les urines liés à des taux élevés d’immunoglobulines de type Ig G (16,82 g/l) avec un taux subnormal des autres classes d’immunoglobulines. Le myélogramme ainsi que le bilan radiologique osseux étaient normaux.Le diagnostic d’une DM associée à une gammapathie monoclonale bénigne était retenu. La patiente était mise sous corticothérapie à forte dose: prednisone à la dose de 1mg/kg/j pendant six semaines avec dégression progressive de 5 mg à chaque semaine jusqu’à une dose d’entretien de 10 mg/j. L’évolution était favorable pour sa DM avec marquée par amélioration progressive des lésions érythémato-oedémateuses et normalisation des enzymes musculaires. Cependant, la patiente développait un diabète cortico-induit traité par des anti-diabétiques oraux. Elle avait un suivi régulier à la consultation externe durant trois années successives et puis perdu de vue avec un recul évolutif de 39 mois.

### Observation 4

Madame D.T âgée de 73 ans, sans antécédents pathologiques notables, était hospitalisée en Octobre 2008 pour l’exploration de lésions érythémateuses touchant le visage notamment au niveau des pommettes, le cuir chevelu, la face antérieure du thorax et la face antérieure des deux genoux évoluant depuis deux mois, compliqué un mois avant son admission par l’apparition de myalgies diffuses, de dysphagie avec modification de la voie devenant nasonnée, fausses routes à répétition le tout évoluant dans un contexte d’altération de l’état général à type d’asthénie, amaigrissement chiffré à huit kilogrammes en deux mois et quinze jours motivant ainsi son hospitalisation. L’examen clinique objectivait la présence d’un érythème télangiectasique qui touche les pommettes, les paupières, les mains notamment en péri- unguéal avec signe de la manicure, le décolleté, le cuir chevelu et la face antérieure des deux genoux. L’examen ostéo-articulaire mettait en évidence un déficit musculaire au niveau de la ceinture scapulaire avec difficulté à la surélévation des deux membres supérieures au delà de 90 degré plus marqué du coté gauche. L’examen oto-rhino laryngé montrait une muqueuse buccale et oro-pharyngée pale, un voile du palais symétrique et mobile, le larynx et l’hypopharynx et le cavum étaient normaux. L’examen cardio-pulmonaire, neurologique, ganglionnaire, digestif et génital était normal.

A la biologie, on notait un taux de leucocytes normal à 9900 éléments/mm3, une hémoglobine à 12,9g/dl, des plaquettes à 255000 éléments/mm3, la vitesse de sédimentation était à 25 mm la première heure, l’électrophorèse des protéines sériques était normale, le bilan rénal était également sans anomalie. Le dosage des transaminases montrait des ASAT à 71UI/l, les ALAT était à 58UI/l, les CPK étaient à 827 UI/l, les LDH à 809UI/l. Le bilan immunologique concluait à un taux d’anticorps anti-nucléaires positif à un taux de 1/640. Les anticorps anti-cytoplasme des polynucléaires neutrophiles étaient négatifs ainsi que les anticorps anti-phospholipides. Devant ces données cliniques et biologiques, le diagnostic de dermatopolymyosite a été évoqué. La patiente avait bénéficié d’un EMG des quatre membres qui s’est révélé normal. Une biopsie cutanéo-muqueuse était faite devant l’aggravation des lésions érythémateuses concluant à un épiderme aminci avec atrophie du corps muqueux, la membrane basale était pigmentée et rectiligne, le derme superficiel contenant un discret infiltrat lympho-histiocytaire, le réseau élastique était focalement détruit. La biopsie neuromusculaire pratiquée au niveau du muscle deltoïde montrait une discrète inégalité des fibres avec présence de fibres nécrotiques à cytoplasme pâle, d’autres sont en voie de résorption macrophagique, les coupes sériées en paraffine montraient des infiltrats inflammatoires lymphoplasmocytaires au niveau des septas et des régions péri vasculaire, il n’avait pas d’atrophie péri fasciculaire, cet aspect histologique était compatible avec le diagnostic d’une DPM. Devant l’altération profonde de l’état général et l’âge avancé, un bilan à la recherche d’une néoplasie profonde était réalisé concluant à des marqueurs tumoraux négatifs, la radiographie thoracique, l’échomammographie, l’échographie abdominopelvienne, la fibroscopie digestive, et l’échographie cervicale étaient sans anomalies. Devant la présence de signe de gravité clinique évoquant une atteinte des muscles laryngo-pharyngés avec dysphagie importante, dysphonie, fausses routes et l’apparition d’un reflux de liquide par le nez, la patiente était traitée par trois bolus de méthylprednisolone relayée par une corticothérapie à forte dose 1mg/kg/jour pendant six semaines avec dégression progressive et maintient d’une dose d’entretient de 20 mg/j de prednisone, cette corticothérapie était associé à un traitement immunosuppresseur: méthotrexate à la dose de 15mg/semaine. L’évolution était marquée par l’aggravation initiale de l’atteinte pharyngée avec dysphagie importante une semaine après le démarrage de la corticothérapie nécessitant la mise en place d’une sonde naso-gastrique durant un mois. On notait également une amélioration progressive des lésions cutanées et des forces musculaires avec normalisation du taux des CPK et LDH. La corticothérapie était compliquée chez cette patiente par l’apparition d’une ostéoporose confirmée par ostéodensitométrie.

Le recul évolutif est de trente quatre mois. La patiente est encore suivie à la consultation externe, le dernier contrôle clinique montrait quelques lésions érythémato-squameuses du visage et du décolleté, absence de déficit moteur des ceintures scapulaires et pelviennes, le taux des CPK était normal (188 UI/l), les LDH étaient légèrement élevées à 577 UI/l.

### Etude descriptive

Parmi les 78 cas de myopathies inflammatoires colligés dans le service durant la période d’étude, 4 patients avaient un âge supérieur ou égal à 65 ans au moment du diagnostic de la maladie, soit une fréquence de 5,12% de toutes les myosites. Il s’agissait d’une dermatomyosite (DM) dans les 4 cas soit une fréquence de 9,09% des DM diagnostiquées (4 parmi 44 cas de DM). L’âge moyen du début de la DM était de 78,5 ans avec des extrêmes allant de 71 à 86 ans. Il s’agissait de 3 femmes et un homme. Le sexe ratio H/F était de 0, 33. La comparaison avec le groupe de DM dont l’âge est < 65 ans ne trouve pas différence significative pour le sexe (p = 0,910). Le délai moyen du diagnostic de la DM était de 2 mois avec des extrêmes allant de 15 jours à 3 mois. Les signes cutanés (érythème et/ou œdème du visage) étaient les plus fréquents retrouvés dans 75% des cas. Les signes généraux étaient présents chez un patient. Il s’agissait d’une asthénie associée à un amaigrissement. L’atteinte musculaire initiale était fréquente (50%). Elle était absente dans les deux autres cas. Les myalgies étaient présentes chez deux patients (50%). Le déficit musculaire proximal était noté à l’examen clinique dans trois cas (75%). Il a intéressé la ceinture scapulaire et pelvienne. L’atteinte des muscles laryngo-pharyngés était fréquente (50%). L’atteinte cutanée était dominée par l’érythème périorbitaire noté dans 4 cas associé à un œdème dans 2 cas. Les autres localisations de l’érythème étaient: péri unguéal dans un cas avec signe de la manicure, décolleté dans 3 cas, faces d’extension des genoux dans 2 cas et cuir chevelu dans un cas. Le syndrome de Raynaud était présent dans un cas, la capillaroscopie objectivait un aspect de microangiopathie organique et présence de mégacapillaires. Des arthralgies inflammatoires des grosses articulations étaient signalées dans un seul cas, elles touchaient les hanches, les genoux et les épaules. Les manifestations digestives étaient notées dans 2 cas à type de dysphagie haute avec fausses routes. Elles étaient inaugurales de la maladie dans les 2 cas. L’atteinte pulmonaire était notée dans un seul cas à type de détresse respiratoire survenue au cours de l’évolution de la maladie.

Un syndrome inflammatoire biologique (SIB) était présent chez 3 patients. La valeur moyenne de la vitesse de sédimentation chez nos 4 malades était de 52,5 mm à la première heure (38 à 80 mm H1).Les enzymes musculaires étaient élevées chez tous nos patients. L’augmentation des CPK était notée dans 4 cas, celle de la LDH dans trois cas. Les transaminases étaient élevées chez 3 patients. Les anticorps antinucléaires étaient recherchés chez tous les patients. Ils étaient positifs chez trois patients.

L’électromyogramme (EMG) était pratiqué dans tous les cas. Il montrait un tracé myositique dans 2 cas, neurogène dans un cas, le tracé était normal dans l’autre cas. La biopsie musculaire était réalisée chez 3 patients. Elle était compatible avec une myosite certaine, l’inflammation et la nécrose étaient les aspects les plus fréquents. L’association à une hypothyroïdie était notée dans un cas. La DM était associée à une néoplasie ovarienne chez une patiente. Elle était d’apparition concomitante. Dans notre série, tous nos patients ont été traités. Les moyens thérapeutiques étaient basés sur les corticoïdes, les immunosuppresseurs et le traitement symptomatique. La corticothérapie était indiquée en première intention chez tous les patients et administrée à forte dose (1mg/Kg/j) d’équivalent Prednisone. Elle était initiée par des bolus de méthylprednisolone à raison de 1g/j trois jours de suite chez 2 patients. Ces bolus étaient indiqués devant la gravité du tableau clinique dans ces 2 cas avec atteinte des muscles laryngo-pharyngés, la dysphagie importante, la dysphonie, les fausses routes et l’apparition d’un reflux de liquide par le nez. Le recours au traitement immunosuppresseur était indiqué chez un seul patient. La molécule était le méthotrexate à la dose de 15 mg/semaine. La durée moyenne du suivi était de 19 mois (2 - 39 mois). L’évolution était défavorable chez une patiente avec installation d’une insuffisance respiratoire aigue nécessitant sa sortie contre avis médical avec décès immédiat. Un patient était perdu de vue après sa sortie de l’hôpital. Les deux autres patients avaient un suivi plus prolongé respectivement de 34 et 39 mois avec une amélioration partielle de leur symptomatologie sous corticothérapie. La morbidité iatrogène due à l’utilisation des corticoïdes a concerné deux patients. Il s’agissait d’un diabète cortico-induit nécessitant la mise du patient sous anti-diabétiques oraux et d’ostéoporose confirmée par ostéodensitométrie pour l’autre patient.

La comparaison entre les deux groupes DM du sujet jeune (âgé de moins de 65 ans) et DM du sujet âgé trouve les résultats suivants: Le délai diagnostique moyen de la DM du sujet âgé était de 2 mois versus 6,1 mois par rapport à la myosite du sujet jeune; le mode de début était essentiellement aigu (p = 0,001); la néoplasie était plus fréquente mais sans différence significative (p = 0,25) (Tableau 1).

**Table 1:** Comparaison dermatomyosite du sujet âgé et dermatomyosite du sujet jeune chez un groupe de patients vues au service de médecine interne du CHU Hèdi Chaker, Tunisie

	Adulte jeune n = 40	Sujet âgé N = 4	*p*
Sexe	9H/31F	1H/3F	0,91
Signes généraux n (%)	22 (55%)	1 (25%)	0,84
Myalgies n(%)	35 (87,5%)	2 (50%)	0,45
Déficit musculaire proximal n(%)	35 (87,5%)	3 (75%)	0,45
Atteinte laryngopharyngée n(%)	21 (52,5%)	2 (50%)	0,61
Signes digestifs n (%)	20 (50%)	2 (50%)	0,60
Signes articulaires n(%)	39 (97,5%)	1 (25%)	0,74
Signes pulmonaires n (%)	7 (17,5%)	1 (25%)	0,36
Néoplasie n(%)	2 (5%)	1 (25%)	0,25
Syndrome inflammatoire biologique	16 (40%)	3 (75%)	0,69
Décès	6 (15%)	1 (25%)	0,53

## Discussion

La DM est une connectivite rare; son incidence annuelle varie de 2 à 10 cas/millions d’habitants selon les populations étudiées [2, 6, 7]. La disparité de l’incidence annuelle selon les pays serait expliquée par des facteurs génétiques et environnementaux [8]. En terme de fréquence tout âge confondu, la DM est la plus fréquente des myopathies inflammatoires notamment chez l’enfant et l’adulte jeune. La survenue de la DM après l’âge de 65 ans est une éventualité rare [5]. La fréquence des myopathies inflammatoires du sujet âgé est difficile à évaluer compte tenu de l’hétérogénéité des populations étudiées [9–11]. Peu d’études se sont intéressées sur les myopathies inflammatoires survenant à un âge tardif [9, 10]. Dans l’étude de Pautas et al [9], colligeant 200 cas de PM/DM, les auteurs trouvent 21 patients qui sont âgés de plus de 65 ans soit une fréquence de 10,5%. Marie et al [10] rapportent une fréquence de 29% (23 cas parmi une série de 79 patients atteints de PM/DM). Dans notre service parmi les 78 sujets suivis pour une myopathie inflammatoire, 4 patients (5,12%) avaient un âge > à 65 ans soit une fréquence moindre que celle rapportée dans la littérature. La DM peut survenir à n’importe quel âge mais elle touche essentiellement l’adulte [12]. Elle possède deux pics de fréquence: entre 5 et 14 ans pour l’enfant et dans la 5ème ou 6ème décennie chez l’adulte [2]. Chez le sujet âgé, la DM reste rare et le plus souvent associée à une pathologie néoplasique [5]. L’âge moyen de nos patients était de 78,5 ans avec des extrêmes allant de 71 à 86 ans versus un âge moyen de 35,4 + 15,4 ans (12-64) dans le groupe sujet jeune. Dans la série de Pautas [9], l’âge moyen des 21 patients âgés était de 69,9+ 4,8 ans (extrêmes de 65 et 80 ans) versus un âge moyen de 46.4 ± 12.4 ans (extrêmes de 23 - 64) pour les patients de moins de 65 ans avec des différences significatives entre les 2 groupes. La DM est plus fréquente chez les femmes que chez les hommes quel que soit l’âge mais cette différence semble s’atténuer chez les sujets les plus âgés.

Une prédominance féminine était constatée chez nos patients (sex ratio femme/homme à 3), ces résultats ont été trouvés dans différentes séries de la littérature [4, 5, 13, 14]. L’installation de la maladie est souvent insidieuse et progressive [15]. Il faut y penser devant des manifestations de faiblesse, de fatigue, d’intolérance à l’effort et ne pas négliger ces symptômes chez les sujets âgés [15]. La DM du sujet âgé se caractérise par un retard diagnostique et une fréquence des formes chroniques [9]. Chez nos patients, le début était aigu avec un délai moyen de diagnostic de 2 mois. L’atteinte musculaire peut inaugurer la maladie, et elle est représentée essentiellement par le déficit musculaire et les myalgies [16]. Le déficit touche typiquement la musculature striée de façon bilatérale, symétrique et non sélective. Il prédomine sur les muscles proximaux notamment sur la ceinture scapulaire et surtout pelvienne et sur les muscles cervicaux. L’intensité de la faiblesse est variable d’u sujet à l’autre [12]. Ce déficit était noté chez 75% de nos patients, sa fréquence varie selon la littérature de 60 à 90% [1, 4, 17]. Les myalgies sont rarement associées au déficit musculaire et peuvent être au premier plan dans les formes aigues. Elles sont spontanées ou provoquées par la palpation. La fréquence des myalgies varie de 25 à 70% selon les séries [18].Dans sa série, Pautas et al [9] trouve que les myalgies sont moins fréquentes chez le sujet âgé (47.5% versus 71.5%, p > 0.05). Dans notre série, les myalgies étaient rapportées dans 50% des cas versus 87,5% chez le sujet plus jeune sans différence significative. Les manifestations cutanées peuvent précéder la myosite. Il s’agit d’un érythro-œdème photosensible, prédominant dans les zones découvertes. L’érythème orbitaire en lunette est typique et pathognomonique de la maladie; il touche les paupières et peut s’étendre aux joues sans atteindre la racine du nez [4, 5]. L’érythème peut toucher également la région péri unguéale donnant ainsi le signe de la manucure évocateur de la maladie [5]. Il s’agit d’un érythème congestif, rouge vif et douloureux à la pression [12]. L’érythème peut s’étendre également au décolleté, face d’extension des genoux, coudes et mains. Dans notre série, l’érythème périorbitaire était noté chez tous nos patients associé à un œdème dans deux cas. L’atteinte digestive est due à l’inflammation de la musculature striée et lisse des différents segments du tube digestif [19]. Les troubles oesopharyngés résultent de l’atteinte de la musculature striée du pharynx et de la partie supérieure de l’œsophage se traduisant par une dysphagie et des fausses routes [20]. Dans la plupart des séries, ainsi que pour nos patients, l’atteinte des muscles oro-pharyngés est précoce et concomitante à l’atteinte des muscles proximaux [21]. L’atteinte oesophagienne est plus fréquente chez les sujets âgés [22].

L’association des MI (PM/DM) à une néoplasie, tout âge confondu, n’est pas fortuite et ce lien entre les deux pathologies est désormais bien établie. Sa fréquence varie entre 6 et 47,8% des cas selon les études [4, 9–11, 23, 24] (Tableau 2). Sigurgeirsson et al ont étudié le risque de cancer sur une cohorte de 788 patients dont 392 DM; le risque relatif de développer un cancer dans le groupe DM était de 2,4 pour les hommes et de 3,04 pour les femmes. Dans cette population, 40% des décès sont secondaires au cancer [25]. Les DM du sujet âgé sont fréquemment associées à une néoplasie profonde; l’âge apparait comme un facteur prédictif de survenue du cancer au cours de cette connectivite [4]. En effet, l’association à une néoplasie est estimée selon les études à 10% avant l’âge 65 ans et peut atteindre jusqu’à 50% des cas après 65 ans [26]. Cette association est plus fréquente au-delà de 45 ans [27]. Selon Stockton, le risque de cancer associé est significatif au cours des DM pour des âges situés entre 45 et 74 ans [28].

**Table 2:** Fréquence des néoplasies associés aux myopathies inflammatoires chez le sujet âgé selon quelques séries de la littérature

Auteurs	Année	Pays	Nombre de cas de néoplasie associé aux myopathies inflammatoires /nombre total de myopathies inflammatoires	Fréquence (%)
Marie et al [10]	1999	France	11/23	47,8
Pautas et al [9]	2000	France	5/21	24
Toumi et al [4]	2009	Tunisie	9/70	12,8
Chen et al [11]	2010	Taiwan	44/348 (âge = 60 ans)	12,6
Notre série	2010	Tunisie	1/4	25

Le diagnostic de néoplasie est dans la majorité des cas concomitant à celui de la myosite ou fait quelques mois plus tard [4]. Le risque est plus élevé au cours de la première année qui suit le diagnostic de la DM et persiste jusqu’à la cinquième année après le diagnostic [4, 28]. Sur le plan biologique, les CPK sont souvent plus élevés et les auto- anticorps plus souvent négatifs dans les DM associées à un cancer que dans les DM isolées [27]. Le type de néoplasie associé aux DM varie selon l’étude et la région géographique du patient. Les cancers gynécologiques notamment le cancer de l’ovaire sont les plus fréquents chez les femmes [29] avec plusieurs cas rapportés dans la littérature sous forme de cas [30–32]. Chez les hommes, il s’agit plutôt de cancer du poumon, digestifs et de lymphomes [33–35]. Chez le sujet âgé et selon la littérature, il existe une nette prédominance du cancer du colon [10] et de l’adénocarcinome rectal [9]. Le Tableau 3 résume quelques observations de DM du sujet âgé associées à une néoplasie rapportée dans la littérature [36–38].

**Table 3:** Résumé de quelques observations de DM du sujet âgé associée à une néoplasie rapportée dans la littérature

Auteurs	Age (ans)	Sexe	Délai diagnostic (DM/néoplasie)	Signes de la DM	Type de néoplasie	Traitement
**Fujita et al**. [36]	72	H	concomitant	Erythème visage/signe de Gottron/déficit musculaireElévation des enzymes Histologie(+)	Cancer pulmonaire non à petites cellules	Corticothérapie (1 mg/Kg/jour) pour la DM Chimiothérapie pour le cancer
**Vesic et al** [37]	66	H	DM survenue 6 mois après le cancer	Atteinte cutanée/Myopathie/Elévation des enzymes /EMG/Histologie (+)	Adénocarcinome rectal avec métastases hépatiques et péritonéales	Pas de traitement
**Kim et al**. [38]	61	F	DM survenue 16 mois avant le cancer	Erythème héliotrope/Déficit musculaire/Elévation enzymatique/	Carcinome épidermoide des amygdales	Corticothérapie et Azathioprine pour la DM Chimiothérapie + Chirurgie + radiothérapie
	64	H	DM survenue 20 mois avant le cancer	Erythème héliotrope/Papule de Gottron/Déficit musculaire/Elévation enzymatique	Carcinome épidermoide des amygdales	Traitement chirurgical
**Notre série**	86	F	Concomitant	Erythème péri-orbitaire/Déficit musculaire/Elévation enzymatique/EMG (+)	Tumeur ovarienne	Corticothérapie 1mg/Kg/jour

F : femme ; H : homme

Ainsi devant une DM du sujet de plus de 40 ans, s’impose un bilan carcinologique répété surtout dans les cinq premières années qui suivent le diagnostic. Ce bilan englobe un examen clinque complet avec les touchers pelviens inclus et systématiquement une radiographie thoracique, une échographie abdomino-pelvienne, une mamaographie chez les femmes, les marqueurs tumoraux (PSA et CA125). Les autres investigations se discuterons en fonction des données de l’examen: un scanner thoraco-abomino-pelvien et/ou une colonoscopie, chez les patients ayant des facteurs prédictifs de néoplasie [4, 29]. On rajoute à ces recommandations, la pratique d’un examen ORL systématique chez un patient provenant d’un pays endémique pour les néoplasies du nasopharynx (Maghreb et Asie), ce d’autant que le pronostic de ces néoplasies semble bon après traitement par la corticothérapie et radiothérapie [35].

Le syndrome inflammatoire biologique (SIB) est fréquent au cours des DM. La vitesse de sédimentation est augmentée de façon modérée chez 50 à 60% des cas [3]. Elle n’est pas corrélée à l’intensité de la maladie et elle n’intervient pas dans le diagnostic. L’anémie et la polynucléose sont également fréquentes [19]. Le SIB était présent chez 3 de nos patients (75% des cas). L’augmentation des enzymes musculaires témoignant de la nécrose musculaire constitue un critère diagnostique [3]. La créatine kinase (CPK) représente l’enzyme musculaire la plus spécifique, elle renseigne sur une souffrance objective du tissu musculaire. Elle est informative si elle est réalisée selon les recommandations d’usage après 24 à 48 heures de repos physique, et significative si les valeurs sont à plus de 2 à 3 fois la normale [15]. Les CPK sont augmentée dans 75 à 85% des cas, cependant un taux normal ne doit pas écarter le diagnostic [3]. Les autres enzymes musculaires sont: l’aldolase sérique qui peut être utile (elle est parfois la seule augmentée), les transaminases et la lacticodéshydrogènase sont moins spécifiques [3, 19]. Chez nos patients, on a noté une élévation des CPK dans tous les cas, les LDH et les transaminases étaient élevées dans trois cas, l’aldolase était élevée chez un patient. Concernant les anomalies immunologiques, la fréquence des anticorps antinucléaires est estimée entre 30 à 50% des cas dans les séries [3]. Dans notre série, les AAN étaient positifs chez trois de nos patients. Ces anomalies biologiques et immunologiques n’ont pas de particularité en fonction de l’âge [15]. La corticothérapie à forte dose (1 mg/kg/j de prednisone) associée aux mesures hygiéno-diététiques usuelles (notamment prévention de l’ostéoporose par les biphosphonates, régime hyposodé, sans sucres rapides et pauvre en graisses saturées), constitue le traitement de première intention des M.I. Avant l’utilisation des corticoïdes, le pronostic des DM était médiocre avec une mortalité de 50 à 60% [39]. La DM est corticosensible, monophasique ou plus souvent chronique. Les corticoïdes constituent le traitement de référence et de première ligne des myosites. 60 à 70% des DM répondent à la corticothérapie orale à base de prednisone [39]. Le mécanisme d’action du prednisone n’et pas clairement bien établi, mais plusieurs hypothèses ont été avancées. Il pourrait inhiber le recrutement et la migration des lymphocytes dans les différents sites inflammatoires, également interférer avec la synthèse des lymphokines notamment l’interleukine 1, Interleukine 2 et le tumor necrosis factor sécrétées par les macrophages activés et les cellules T [40]. Les corticoïdes sont prescrits à la dose de 1mg/kg/j d’équivalent de prednisone précocement au cours de l’évolution de la maladie. Ces fortes doses sont maintenus pendant 4 à 8 semaines en moyenne, jusqu’à la régression des signes cliniques et la nette diminution ou la normalisation des enzymes musculaires; une dégression progressive peut être par la suite entamée jusqu’à l’obtention de la dose minimale efficace qui sera maintenue pendant 6 à 9 mois sans rechutes [41]. L’utilisation de bolus intraveineux de Méthyl-Prednisolone à la dose de 1g kg j pendant 3j relayé par une corticothérapie forte dose est réservée aux formes sévères de DM [39]. Dans sa forme paranéoplasique, le traitement de la tumeur peut très rarement être à lui seul curatif. La corticothérapie est quasiment toujours nécessaire; la rechute de la DM fait suspecter celle du cancer [2]. Plusieurs facteurs sont associés à une faible réponse au corticoïdes, notamment: le retard à l’instauration des corticoïdes, l’âge avancé, l’atteinte interstitielle pulmonaire et l’atteinte cardiaque, l’association à une néoplasie, la détection d’anticorps de type anti-synthétase et anti-SRP [42]. Pautas et al. n’ont pas trouvé de différence concernant le traitement entre les 2 groupes sujet âgé/sujet jeune, en dehors de la durée du traitement qui était plus longue dans le second groupe (p = 0.008) [9]. Les effets secondaires de la corticothérapie étaient plus fréquents chez les sujets âgés. En cas de myosite réfractaire ou corticodépendante, aussi bien pour le sujet jeune que pour le sujet âgé, d’autres options thérapeutiques sont utilisées incluant le methotrexate (MTX), l’azathioprine, le mycophénolate mofetil, les immunoglobulines intraveineuses et le rituximab [9]. On recommande généralement le repos en période de maladie évolutive et d′attendre que les symptômes soient maîtrisés avant de faire de l′exercice pour éviter la perte de tonus musculaire. La prévention des pneumopathies d′inhalation, la kinésithérapie (passive et douce lors des poussées inflammatoires, active et régulière dès les premières semaines de corticothérapie passées, jusqu’à la fin de la corticothérapie) et l′ergothérapie sont indispensables dans la prise en charge de ces patients. La kinésithérapie et la réadaptation musculaire sont absolument indispensables, favorisant le trophisme musculaire, luttant contre la myopathie stéroïdienne et améliorant la vascularisation myocytaire. Lors du retour à domicile après hospitalisation, il est souhaitable que tout soit bien organisé pour recevoir le sujet âgé encore fatigué et souvent sous traitement en évaluant son autonomie et son indépendance après cette pathologie inflammatoire musculaire.

## Conclusion

La DM, bien que rare chez la personne âgée, ne doit pas être méconnue et doit faire rechercher une pathologie cancéreuse sous-jacente. Notre étude constitue le premier travail réalisé en Tunisie concernant les DM du sujet âgé. Elle nous a permis de mieux connaître les caractéristiques de cette forme et de la comparer aux différentes séries de la littérature. Malgré le traitement intensif par corticoïdes associées parfois à des immunosuppresseurs, ces DM sont grevées d’une importante morbidité et mortalité qui alourdissent le pronostic de la maladie.
